# Sodium pentobarbital dosages for exsanguination affect biochemical, molecular and histological measurements in rats

**DOI:** 10.1038/s41598-019-57252-7

**Published:** 2020-01-15

**Authors:** Ayman S. Mohamed, Mohamed Hosney, Heba Bassiony, Sarah S. Hassanein, Amel M. Soliman, Sohair R. Fahmy, Khadiga Gaafar

**Affiliations:** Department of Zoology, Faculty of Science, Cairo University, Giza, 12613 Egypt

**Keywords:** Molecular biology, Animal physiology, Cell biology, Chemical biology

## Abstract

Rodents are widely used for animal research in Egypt. Pentobarbital is the most common anesthetic agent; however overdoses may affect the experimental outcomes and limit the use of tissues. To investigate the effects of sodium pentobarbital overdoses during exsanguination, three groups (6 rats/group) of male and female rats were injected i.p. with 50, 100 and 150 mg/kg of sodium pentobarbital, then carotid exsanguination was performed immediately after loss of consciousness. Hypoxia-inducible factor 1-alpha (Hif1a) and tumor necrosis factor-alpha (Tnfa) mRNA expressions in liver and kidney organs were evaluated. As well as, serum aminotransferase activities (AST&ALT), glucose, urea, creatinine, malondialdehyde (MDA), reduced glutathione (GSH) and catalase (CAT) levels were determined. The histological alterations in liver, kidney and spleen were studied. It was found that Hif1a and Tnfa were significantly overexpressed in the studied organs and serum AST, glucose, creatinine and urea levels were significantly increased after sodium pentobarbital overdoses (100 and 150 mg/kg) compared to 50 mg/kg dose. Similarly, significant increase in MDA and GSH levels of liver, kidney and spleen were noticed. Results showed gender difference where Hif1a and Tnfa levels were significantly overexpressed at high dose of sodium pentobarbital of liver and kidney organs in female more than male rats. Since euthanasia protocol may influence the physiological variables and affect genes’ expression, it is recommended to avoid sodium pentobarbital overdose during euthanasia as it may interfere with the biochemical, molecular and histological measurements.

## Introduction

In Egypt, rodents are considered the first choice for the majority of animal research and training. Euthanasia of animals is generally performed upon completion of the study or if a humane endpoint has been reached. Last updates of the Canadian Council on Animal Care (CCAC) and American Veterinary Medical Association (AVMA) euthanasia guidelines^1^ describe several techniques for successful euthanasia to refine killing methods for rats and other laboratory animals^2^.

Euthanasia procedures are divided into two main methods; chemical and physical. Ideally, a mechanical method, such as exsanguination, should be implemented after the chemical death. Pentobarbital is an anesthetic agent commonly used in euthanasia. Olsen and Li (2011) reported that sodium pentobarbital is an injectable, fast-acting, central nervous system depressant which acts via GABA receptors to cause a loss of consciousness and cardiovascular depression^3^. In the AVMA Guidelines, anesthetic overdose is recommended as an acceptable method of euthanasia. For rodents, overdose of sodium pentobarbital is the most preferable euthanizing agent^4–6^.

The main reported negative side effects of pentobarbital mono-anesthesia are cardiovascular and respiratory systems depression, decreased arterial blood pressure, peripheral vasodilation, decreased cardiac output and depression of the vasopressor response to hemorrhage^7^. Moreover, the effects of anesthesia on metabolism have been studied previously and are the topic of several reviews^8,9^. Arnold and Langhans (2010) have focused on understanding the impact of short-term anesthesia or euthanasia as a step in tissue collection procedures for rodents^10^. In addition, the molecular basis of anesthetic action has been studied^11,12^. The principle molecular targets of different anesthetics are cellular entities in the heart, liver, and peripheral vasculature^13^.

Existing guidelines do not indicate a specific dose of sodium pentobarbital that is needed to exert deep anesthesia in rodents while performing exsanguination as a secondary physical method of euthanasia. Although 150 mg/kg or 3 times the anesthetic dose has been suggested^14^, the metabolic, molecular as well as histological implications of such doses were not taken into consideration. However, the use of sodium pentobarbital overdose may affect the experimental outcomes which may limit the use of the tissues from the euthanized animal. In an attempt to specify the optimum dosage of sodium pentobarbital before performing exsanguination in rats, the present investigation was designed to study the biochemical, molecular and histological effects of three different dosages of sodium pentobarbital for euthanasia in rats.

## Results

### Expression of Hif1a and Tnfa in liver and kidney organs

**Figure 1:**
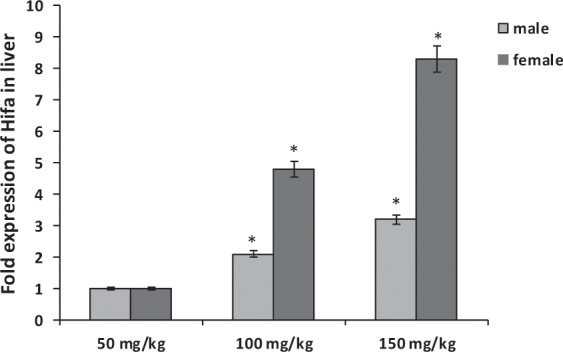
Comparison of the effect of sodium pentobarbital (50, 100 and 150 mg/kg) on the expression levels of Hif1a mRNA in liver of males and females rats. *Statistically significant at P < 0.001 compared to the rats treated by 50 mg/kg of sodium pentobarbital group.

**Figure 2:**
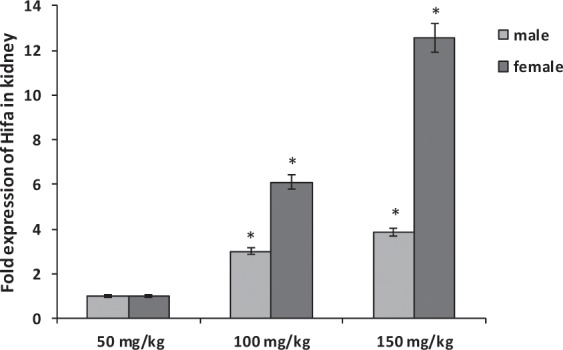
Comparison of the effect of sodium pentobarbital (50, 100 and 150 mg/kg) on the expression levels of Hif1a mRNA in kidney of males and females rats. *Statistically significant at P < 0.001 compared to the rats treated by 50 mg/kg of sodium pentobarbital group.

**Figure 3:**
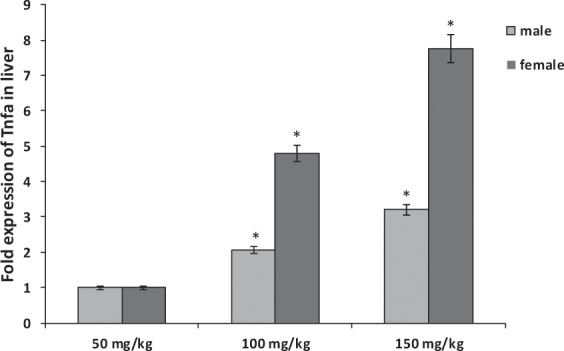
Comparison of the effect of sodium pentobarbital (50, 100 and 150 mg/kg) on the expression levels of Tnfa mRNA in liver of males and females rats. *Statistically significant at P < 0.001 compared to the rats treated by 50 mg/kg of sodium pentobarbital group.

**Figure 4:**
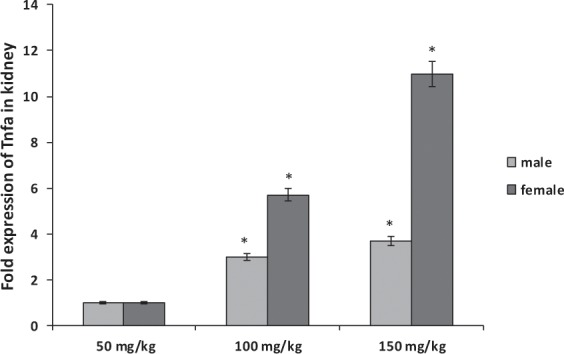
Comparison of the effect of sodium pentobarbital (50, 100 and 150 mg/kg) on the expression levels of Tnfa mRNA in kidney of males and females rats. *Statistically significant at P < 0.001 compared to the rats treated by 50 mg/kg of sodium pentobarbital group.

The mRNA expression levels of Hif1a, and Tnfa genes in the liver and kidney organs of male and female rats were assessed and changes were shown in Figs. (1–4). The fold expression of Hif1a, and Tnfa genes in the groups that were treated with 100 and 150 mg/kg of sodium pentobarbital was compared with 50 mg/kg treated group. On the whole, results showed that Hif1a levels were significantly overexpressed in liver and kidney organs treated with 150 mg/kg of sodium pentobarbital than those treated with 100 mg/kg and 50 mg/kg of sodium pentobarbital in both male and female rats. On the other hand, rats treated with dosage 100 and 150 mg/kg sodium pentobarbital i.p. were found to exhibit increased levels of Hif1a in liver organ in males by 2.1-fold and 3.2-fold respectively, while in females by 4.8-fold and 8.3-fold, respectively (P < 0.001; Fig. 1). Concomitantly, in the kidney organ, the Hif1a increased in expression in males by 3-fold and 3.85-fold, respectively, and by 6.1-fold and 12.55-fold, respectively for females (P < 0.001; Fig. 2).

In addition, the levels of Tnfa were significantly overexpressed in liver and kidney organs of male and female rats treated by 150 mg/kg of sodium pentobarbital than in those treated by 100 mg/kg and 50 mg/kg. Also gender difference was observed in liver organ Hif1a of rats treated with dosage 100 and 150 mg/kg sodium pentobarbital i.p: in males by 2.05-fold and 3.2-fold, and in females by 4.8-fold and 7.75-fold, respectively (P < 0.001; Fig. 3).While the increasing folds in kidney organ were 3-fold and 3.7-fold, respectively in males, and 5.7-fold and 10.95-fold, respectively in females (P < 0.001; Fig. 4). Thus the fold expression of both genes (Hifla and Tnfa) in liver and kidney organs was more increased at higher dosage of sodium pentobarbital in females than in male rats.

### Histological examination of liver, kidney, and spleen organs

**Figure 5:**
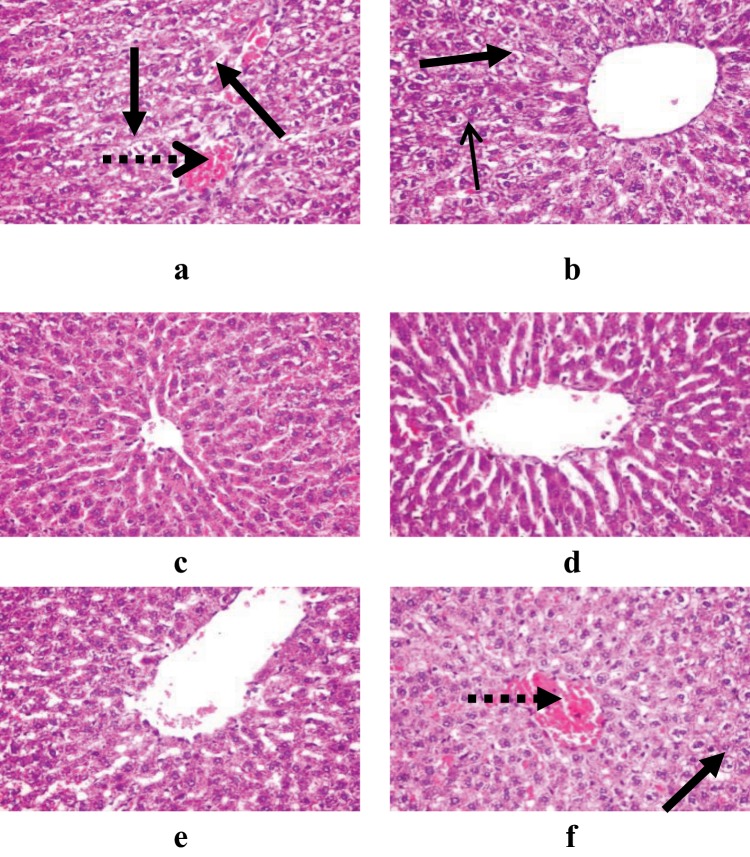
Demonstrating histopathological examination of liver in male and female rats treated by 50 mg/kg (**a,b**), 100 mg/kg (**c,d**) and 150 mg/kg (**e,f**) sodium pentobarbital (H&E X 400). Long dotted arrows: congested central vein and long solid arrows: scattered vacuolated hepatocytes.

Examination of the liver sections (Fig. 5), it was observed that the lower dose (50 mg/kg) of sodium pentobarbital showed congested central vein and scattered vacuolated hepatocytes in male rats, while only scattered vacuolated hepatocytes in female rats were observed. The higher dose (150 mg/kg) of sodium pentobarbital showed a healthy hepatic parenchyma, hepatocytes and more leucocytic cells infiltrations in portal area in liver organ of both male and female rats than that treated by 100 mg/kg sodium pentobarbital.

**Figure 6:**
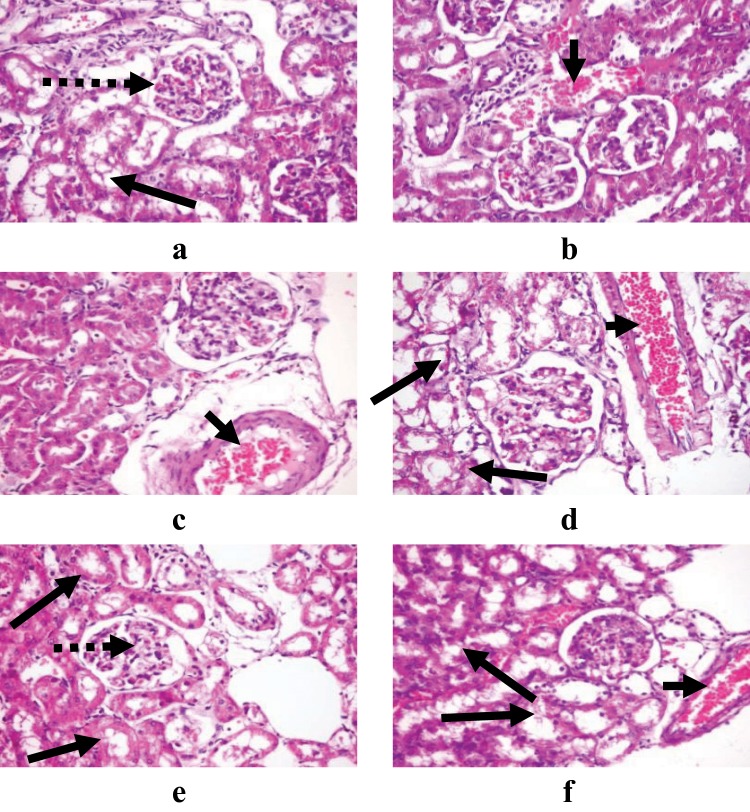
Demonstrating histopathological examination of kidney in male and female rats treated by 50 mg/kg (**a**,**b**), 100 mg/kg (**c**,**d**) and 150 mg/kg (**e**,**f**) of sodium pentobarbital (H&E X 400). Long dotted arrows: congested glomerular tuft capillaries, long solid arrows: vacuolated renal tubular epithelium, and short solid arrows: congested interstitial blood vessel.

Examination of the kidney sections (Fig. 6), the lower dose (50 mg/kg) of sodium pentobarbital showed normal glomeruli and renal tubules in male rats and a congested interstitial blood vessel in female rats. While, congested interstitial blood vessel was observed in male and female rats treated by 100 mg/kg sodium pentobarbital. Furthermore, congested glomerular tuft capillaries and vacuolated renal tubular epithelium were found in kidney organ of male rats treated by 150 mg/kg sodium pentobarbital. Otherwise, congested interstitial blood vessel and degenerated renal tubules were noticed in female rats treated by 150 mg/kg sodium pentobarbital than that treated by 100 mg/kg sodium pentobarbital.

**Figure 7:**
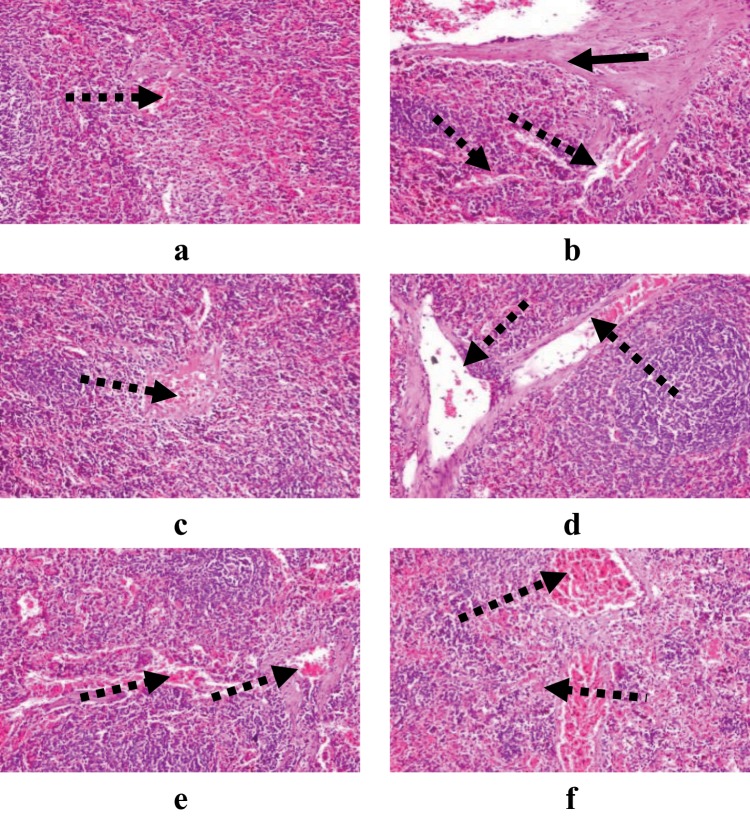
Demonstrating histopathological examination of spleen in male and female rats treated by 50 mg/kg (**a**,**b**), 100 mg/kg (**c**,**d**) and 150 mg/kg (**e**,**f**) sodium pentobarbital (H&E X 200). Long dotted arrows: congested blood vessel, and long solid arrows: fibrous connective tissue proliferation.

Examination of the spleen sections in both male and female rats groups (Fig. 7), showed no marked difference among the three treated groups, only congested blood vessel was observed.

Therefore, it was concluded that liver and kidney organs for both male and female rats were more affected by the higher the dose. In addition, spleen was the least affected organ in both male and female rats by the three different doses of sodium pentobarbital.

### Immunohistochemical findings

**Figure 8:**
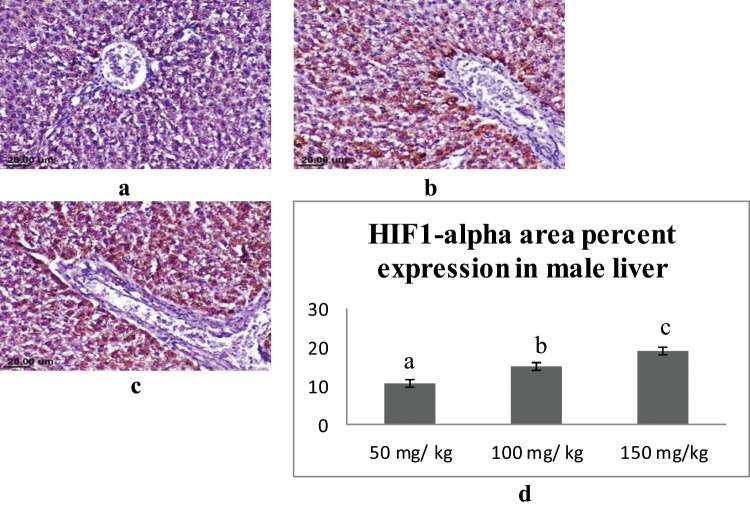
IHC for HIF1-alpha in liver sections from male rats; (**a**) male rat receiving 50 mg/kg, (**b**) male rat receiving 100 mg/kg and (**c**) male rat receiving 150 mg/kg. (**d**) Area percent expression of HIF1-alpha of different groups, error bars represent the standard error of the mean (n = 15), a, b and c above the error bar indicate a significant difference between values within the same data series.

**Figure 9:**
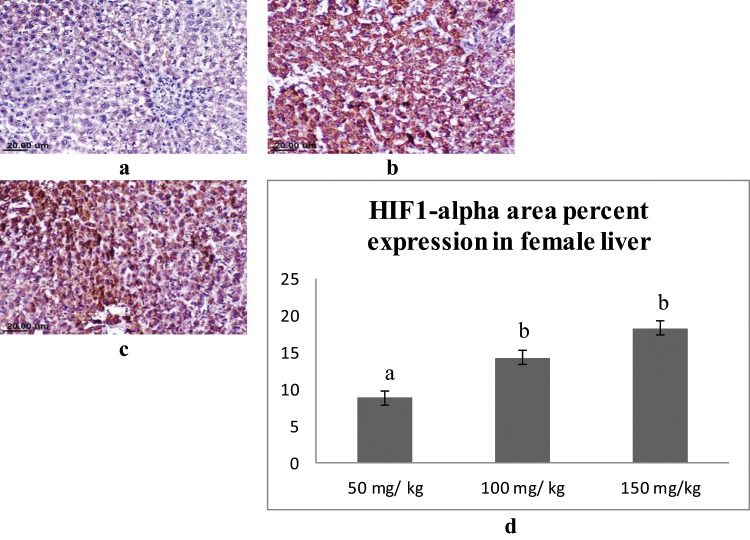
IHC for HIF1-alpha in liver sections from female rats; (**a**) female rat receiving 50 mg/kg, (**b**) female rat receiving 100 mg/kg and (**c**) female rat receiving 150 mg/kg. (**d**) Area percent expression of HIF1-alpha of different groups, error bars represent the standard error of the mean (n = 15), a and b above the error bar indicate a significant difference between values within the same data series.

Immunohistochemical staining was performed for semi-quantitative assessment of the percent area expression of HIF1-alpha cellular proteins in liver and kidney organs of male and female rats. Liver sections of male rats from different groups showed varied positive reaction. Expression of HIF1-alpha was strongly detected in the hepatocytes of the male rats administrated 150 mg/kg sodium pentobarbital than 100 mg/kg sodium pentobarbital (19.12% and 15.09% respectively) compared to dose 50 mg/kg sodium pentobarbital (10.91%) (Fig. 8). Regarding female rats, the expression of HIF1-alpha was strongly detected in the hepatocytes of the female rats administrated 150 mg/kg than 100 mg/kg sodium pentobarbital (18.35% and 14.37% respectively) compared to dose 50 mg/kg sodium pentobarbital (8.86%) (Fig. 9).

**Figure 10:**
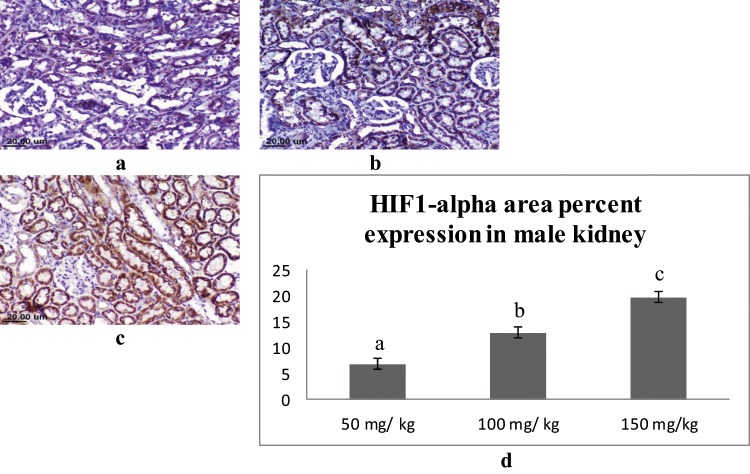
IHC for HIF1-alpha in kidney sections from male rats; (**a**) male rat receiving 50 mg/kg, (**b**) male rat receiving 100 mg/kg and (**c**) male rat receiving 150 mg/kg. (**d)** Area percent expression of HIF1-alpha of different groups, error bars represent the standard error of the mean (n = 15), a, b and c above the error bar indicate a significant difference between values within the same data series.

**Figure 11:**
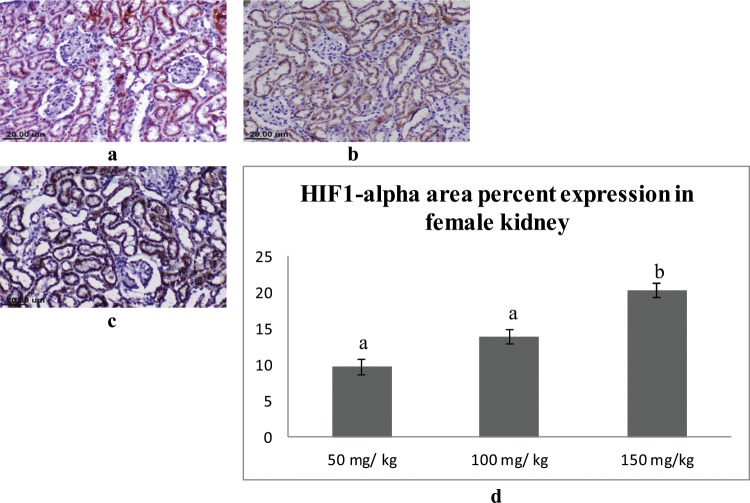
IHC for HIF1-alpha in Kidney sections from female rats; (**a**) female rat receiving 50 mg/kg, (**b**) female rat receiving 100 mg/kg and (**c**) female rat receiving 150 mg/kg. (**d**) Area percent expression of HIF1-alpha of different groups, error bars represent the standard error of the mean (n = 15), a and b above the error bar indicate a significant difference between values within the same data series.

In the kidney, the expression of the HIF1-alpha was mainly detected in the epithelial lining the renal tubules in the cortex and medulla. Male rats that received 150 mg/kg of sodium pentobarbital showed the highest expression (19.77%), followed by 100 mg/kg sodium pentobarbital (12.93%), then 50 mg/kg sodium pentobarbital (6.89%) (Fig. 10). The female rats that received 150 mg/kg showed the highest expression of HIF1-alpha (20.33%) followed by 100 mg/kg sodium pentobarbital (13.93%) then 50 mg/kg sodium pentobarbital (9.68%) (Fig. 11).

### Effect of sodium pentobarbital on some serum biomarkers of rats

Table 1 shows that overdoses of intraperitoneal injection of sodium pentobarbital for euthanasia at dosages of 100 and 150 mg/kg caused a significant increase (P < 0.05) in the AST activity of both male and female rats as compared to the corresponding ones of rats euthanized by the lower dose of sodium pentobarbital (50 mg/kg, i.p). On the other hand, ALT activity of both male and female rats was non–significantly changed in all the dosages of sodium pentobarbital (P > 0.05).

**Table 1:** Levels of some biochemical markers in the serum of male and female rats euthanized by exsanguinations following exposure to different doses of sodium pentobarbital.

Gender	Dose (mg/Kg. body weight)	AST (U/mL)	ALT (U/mL)	Glucose (mg/dL)	Creatinine (mg/dl)	Urea (mg/dL)
	0	—	—	57.75 + 2.13^a^	—	—
Female	50	14.41 ± 2.75^a^	21.46 ± 1.88^a^	60.97 ± 5.18^a^	0.61 ± 0.19^a^	49.61 ± 14.40^a^
	100	17.79 ± 1.44^b^	24.72 ± 3.17^a^	84.82 ± 3.89^b^	0.88 ± 0.21^b^	66.67 ± 14.97^b^
	150	24.59 ± 6.00^b^	25.51 ± 2.45^a^	98.13 ± 8.89^b^	0.91 ± 0.18^b^	70.56 ± 9.70^b^
Male	0	—	—	65.37 + 3.48^a^	—	—
	50	10.25 ± 5.18^a^	22.11 ± 6.28^a^	87.57 ± 8.08^b^	0.59 ± 0.31^a^	52.34 ± 4.96^a^
	100	20.49 ± 4.42^b^	23.86 ± 5.83^a^	101.63 ± 6.54^c^	0.82 ± 0.18^b^	59.67 ± 2.31^b^
	150	21.61 ± 5.32^b^	24.19 ± 4.48^a^	119.11 ± 21.68^c^	0.82 ± 0.36^b^	60.74 ± 8.00^b^
Effect of gender		P > 0.05	P > 0.05	P > 0.05	P > 0.05	P > 0.05
Effect of Dosage		P < 0.05	P > 0.05	P < 0.05	P < 0.05	P < 0.05
Interaction of gender and dosage		P > 0.05	P > 0.05	P > 0.05	P > 0.05	P > 0.05

Values are mean ± SEM (n = 6).

Values within each gender and biochemical marker, different superscripted letters indicate significantly (P < 0.05) different values.

Serum glucose, creatinine and urea levels of male and female rats were significantly increased (P < 0.05) after intraperitoneal injection of sodium pentobarbital for euthanasia at dosages of 100 and 150 mg/kg as compared to the corresponding ones of rats euthanized by sodium pentobarbital (50 mg/kg, i.p) (Table 1). Data recorded in Table 1 revealed non–significant change (P > 0.05) in the levels of the studied serum biomarkers regarding the gender.

### Effect of sodium pentobarbital on oxidative stress biomarkers of rats

Significant increase (P < 0.05) was noticed in the MDA and GSH levels of liver, kidney and spleen of male and female rats euthanized by sodium pentobarbital at high dosages (100 and 150 mg/kg, i.p) as compared to the corresponding ones of rats euthanized by sodium pentobarbital (50 mg/kg, i.p) (Table 2).

**Table 2:** Levels of some oxidative markers in the liver, kidney and spleen of male and female rats euthanized by exsanguinations following exposure to different doses of sodium pentobarbital.

Gender	Organ	Dosage (mg/Kg. body weight)	MDA (nmol/g. tissue)	GSH (mg/g. tissue)	CAT (U/g. tissue)
Female	Liver	50	5.48 ± 0.66 ^a^	6.24 ± 0.88^a^	0.97 ± 0.07^a^
		100	6.71 ± 0.50^b^	8.98 ± 0.62^b^	1.12 ± 0.21^a^
		150	8.43 ± 1.88^c^	12.21 ± 0.25^c^	1.23 ± 0.07^a^
	Kidney	50	3.28 ± 0.83^a^	2.34 ± 0.23^a^	1.01 ± 0.15^a^
		100	4.76 ± 0.54^b^	3.11 ± 0.63^b^	0.97 ± 0.11^a^
		150	7.96 ± 0.58^b^	3.29 ± 0.82^b^	1.26 ± 0.05^a^
	Spleen	50	4.84 ± 0.73^a^	3.31 ± 0.56^a^	0.65 ± 0.22^a^
		100	5.99 ± 1.04^b^	4.66 ± 0.35^b^	0.73 ± 0.06^a^
		150	6.43 ± 0.71^b^	4.99 ± 0.22^b^	0.85 ± 0.10^a^
Male	Liver	50	4.33 ± 0.90^a^	4.12 ± 0.99^a^	1.10 ± 0.13^a^
		100	5.99 ± 1.43^b^	7.14 ± 0.43^b^	1.20 ± 0.15^a^
		150	7.81 ± 0.50^c^	10.75 ± 0.78^c^	1.29 ± 0.04^a^
	Kidney	50	3.97 ± 0.57^a^	2.69 ± 0.84^a^	0.79 ± 0.11^a^
		100	5.33 ± 0.67^b^	3.12 ± 0.60^b^	0.89 ± 0.08^a^
		150	6.47 ± 0.59^b^	3.63 ± 0.02^b^	0.92 ± 0.14^a^
	Spleen	50	4.80 ± 0.55^a^	3.54 ± 0.62^a^	0.79 ± 0.21^a^
		100	5.38 ± 0.41^b^	4.58 ± 0.26^b^	0.96 ± 0.13^a^
		150	5.67 ± 0.20^b^	4.61 ± 0.69^b^	1.01 ± 0.05^a^
Effect of gender		Liver	P > 0.05	P > 0.05	P > 0.05
		Kidney	P > 0.05	P > 0.05	P > 0.05
		Spleen	P > 0.05	P > 0.05	P > 0.05
Effect of Dosage		Liver	P < 0.05	P < 0.05	P > 0.05
		Kidney	P < 0.05	P < 0.05	P > 0.05
		Spleen	P < 0.05	P < 0.05	P > 0.05
Interaction of gender and dosage		Liver	P > 0.05	P > 0.05	P > 0.05
		Kidney	P > 0.05	P > 0.05	P > 0.05
		Spleen	P > 0.05	P > 0.05	P > 0.05

Values are mean ± SEM (n = 6).

Values within each gender and biochemical marker, different superscripted letters indicate significantly (P < 0.05) different values.

Liver, kidney and spleen catalase activity of male and female rats were non-significantly changed (P > 0.05) after euthanization by sodium pentobarbital at high dosages (100 and 150 mg/kg, i.p) as compared to the corresponding ones of rats euthanized by sodium pentobarbital (50 mg/kg, i.p) (Table 2).

Regarding the effect of gender, the liver, kidney and spleen levels of MDA, GSH and CAT were non-significantly changed (P > 0.05) between male and female groups (Table 2).

## Discussion

Euthanasia is an important procedure performed during animal experimentation that ideally should be carried out with the least possible influence on the study parameters. Exsanguination is one of the physical methods of euthanasia with rapid relief of pain and suffering and must be done under anesthesia. Sodium pentobarbital is the most common anesthetic agent used before rodent’s euthanasia by exsanguination^15^. As reported in several current literatures, sodium pentobarbital is used with different dosages which may impact the study parameters^16,17^. Thereby, the present study aimed to specify the optimum dosage of sodium pentobarbital that can be used while performing exsanguination with the least adverse effects on biochemical, molecular and histological measurements.

It has been reported that anesthetic agents often used in studies with laboratory animals significantly alter physiological parameters^18^. Thereby, the choice of the anesthetic agent has to be made according to the experimental protocol and the variables intended to be measured. Pentobarbital is a short-acting sedative and hypnotic agent that produces dose-dependent respiratory depression^19^. In addition, pentobarbital has narrow therapeutic index where the anesthetic dose is close to the dose causing respiratory arrest^20^. It is a known fact that anticonvulsant and hypotensive properties are most frequently associated with pentobarbital overdoses^20^. Results from previous study showed marked hypoxia with high doses of pentobarbital^21^. In addition, barbiturates induced hypoxia was confirmed immediately 5–10 minutes after initiation of anesthesia^22^.

During hypoxia, the living body possesses several homeostatic mechanisms. The response to hypoxia is mainly dependent on oxygen sensing and subsequent adaptation^23^. Changes in gene expression are the first adaptation that is associated with several oxygen-dependent transcription factors. For that, we investigated the expression level of the Hypoxia-inducible factor 1-alpha (Hif1a) and the inflammatory mediator tumor necrosis factor-alpha (Tnfa). As previously reported, TNF-α enhances HIF-1α protein and mRNA levels, under both normoxia and hypoxia conditions^22,24^. The hypoxia-inducible factors (HIFs) are a family of transcriptional regulators of a homeostatic transcriptional response to hypoxia in virtually all cells and tissues. It has been reported that mammalian cells initiate several survival processes such as activation of HIF-1α when subjected to hypoxia^25,26^. A previous study has proposed a mechanism for TNF-α induced HIF-1α translation; TNF-α signals via NFкB, PI3k and MAPK pathways that collectively contribute to Bcl-2 expression which in turn promotes an internal ribosome entry site (IRES)-dependent translation of HIF-1α mRNA^27^.

The present study showed for the first time, to the authors’ knowledge, that Hif1a mRNA expression was involved in the pathogenesis of sodium pentobarbital overdoses (100 and 150 mg/kg) in the liver and kidney of rats. In agreement with previous reports^28,29^, the present study showed that induction of Hif1a mRNA in the liver and kidney can be deemed as a useful index of the severity of hypoxia in rats exposed to pentobarbital overdoses. Moreover, the induction of Hif1a mRNA in the liver and kidney in the present investigation following sodium pentobarbital overdoses (100 and 150 mg/kg) may be due to oxidative stress (OXS), which was manifested by the observed correlation between the values of Hif1a mRNA and the increase of OXS markers.

Interestingly, a gender difference where an increase in the fold expression in female rats of Hif1a and Tnfa mRNA was observed. Similarly, a previous study reported a sex-related dimorphic response of HIF-1α expression in myocardial ischemia, where increased HIF-1α expression in hearts from females both under normoxic and, more strikingly, under hypoxic conditions. They hypothesized a mechanism for that including differential activation of prolyl hydroxylase domain or ubiquitin ligase enzymes or expression of a HIF-1α variant, which might result in a more stable protein^30^. Also, another recent study found higher basal HIF-1α in female human pulmonary artery smooth muscle cells (hPASMCs) compared with male and increased HIF-1α protein expression in female rat whole lung tissue with chronic hypoxia^31^.

Regarding TNF-α, it was found that chronic inflammatory diseases may have different outcomes linked to the gender. Women present higher disease activity markers due to hormonal, genetic and environmental factors^32–34^.

The present study also was extended to investigate the effect of pentobarbital anesthesia on the liver pathogenesis. During pathogenesis of liver injury, the determination of enzyme levels such as serum aminotransferases, AST and ALT is usually used for accurate diagnosis. The present investigation confirmed the explanation of Johnson *et al*., Norman *et al*. and Lin *et al*.^35–37^ who reported that, the elevation in the serum enzymes AST and ALT in response to the induction of Hif1a mRNA expression in the liver organ following sodium pentobarbital overdoses (100 and 150 mg/kg) may be subsequent to hypoxia. These findings are further confirmed by the histological results; the high dose of sodium pentobarbital (150 mg/kg) could induce considerable hepatocellular and renal injury in both male and female rats more than that induced by 100 mg/kg as compared to the lower dose (50 mg/kg). This hepatotoxic effect was previously observed in male rats anesthetized by 1% sodium pentobarbital; those exhibited hepatocytes hypertrophy, swollen, ballooned lipid laden hepatocytes and dilated sinusoidal spaces in most regions, revealing extensive liver lesions^38^. Moreover, the elevation of liver enzymes in the present study may be due to an imbalance between hepatic oxygen supply and demand^39^.

Several attempts have been made to reveal the mechanism of hyperglycemia induction subsequent to anesthesia in rats. Previous studies have indicated that most anesthetic agents induced hyperglycemia except sodium pentobarbital^40–42^. However, other previous studies reported hyperglycemia as a result of pentobarbital overdose (100 and 150 mg/kg) in agreement with the present study^43,44^. The increased glucose plasma levels recorded in the present study may be due to induction of hepatic insulin resistance which in turn causes gluconeogenesis and hyperglycemia^45,46^. Moreover, hyperglycemia following sodium pentobarbital overdoses (100 and 150 mg/kg) anesthesia might be due to modulation of glucose and lipid metabolism^41,47,48^. In addition, higher plasma glucose concentrations in the present investigation most likely resulted from elevated stress levels and hemodilution after resorption of intraperitoneally administered liquids^49^. As well as, it was reported that, the high blood glucose levels lead to induction of oxidative stress^50^.

Oxidative stress during sodium pentobarbital toxicity might be a systemic phenomenon^38^. During animal experimentation, it is imperative to choose the means of euthanasia on the basis of protocol requirement for tissue collection or analysis. In particular, generation of oxygen free radicals and lipid peroxidation play a key role in the development of sodium pentobarbital toxicity. The present study confirmed the finding of Zhang *et al*.^51^ that elevations of lipid peroxidation, malondialdehyde (MDA) in the hepatic, renal and spleen tissues of male and female rats following i.p. injection of sodium pentobarbital (100 and 150 mg/kg) as compared to minimum dosage (50 mg/kg) may reflect the free radical mediated cell membrane damage. The underlying mechanisms of increased tissues oxidative stress during sodium pentobarbital anesthesia may be due to the generation of reactive oxygen species (ROS) which stimulate lipid peroxidation^38^. The current study showed that gender has no effect regarding the level of MDA in the studied tissues.

Hyperglycemia and oxidative stress induce release of inflammatory cytokines, such as tumor necrosis factor-alpha (TNF-α)^52^. TNF-α is considered one of the most important cytokines recognized as a major effector of macrophage-mediated host defense and tissue injury^53^. The key finding of the present study is that the euthanasia protocol may influence physiological variables, which in turn may affect mRNA expression levels. The present study showed that a few minutes of sodium pentobarbital overdoses (100 and 150 mg/kg) anesthesia before euthanasia and tissue sampling are sufficient to induce immediate hepatic and renal Tnfa mRNA dose dependent expression.

Reduced glutathione (GSH) is the main component of the endogenous non-protein sulfhydryl pool that is known to be a major low molecular weight scavenger of free radicals in the cytoplasm^54^. GSH shows a protective effect by neutralizing free radicals and reactive oxygen intermediates. The endogenous anti-oxidant enzymes CAT, SOD, and GST in the liver tissues are the key components of cellular defense system against ROS^55^. In accordance with the report of Torres *et al*.^56^ our results support the notion that exposure to sodium pentobarbital overdoses (100 and 150 mg/kg) for few minutes anesthesia is sufficient to present alteration in the enzymatic antioxidant defense mechanism, showing a disturbance in hepatic, renal and splenic oxidative status, which could contribute to their damage. This increase in antioxidant defense may be due to enhanced oxygen free-radical production, which could stimulate antioxidant activities to cope with increased OXS and protect cells from damage. Moreover, the significant increase of GSH level may suggest its involvement in facilitating the metabolism of the hepatic, renal and splenic cells.

In conclusion, the present study clearly indicated that Hif1a and Tnfa genes expressions were involved in the pathogenesis of sodium pentobarbital overdoses (100 and 150 mg/kg) anesthesia in the liver and kidney of rats but not in the spleen. The recorded results showed that hyperglycemia was the most remarkable metabolic disorder of sodium pentobarbital higher doses which was attributed to induction of hepatic insulin resistance. Overall, oxidative stress during sodium pentobarbital toxicity might be a systemic phenomenon. The underlying mechanisms of increased tissues oxidative stress during sodium pentobarbital anesthesia may be due to lipid peroxidation. Moreover, enzymatic findings and histological changes could be important signs of toxic effects of sodium pentobarbital high dosage administrations. The key finding of the present study is that the euthanasia protocol may influence physiological variables, which in turn may affect mRNA expression level. Therefore, the reasonable selection and control of anesthetics are very important in order to avoid the experimental errors caused by anesthesia. The main recommendation of the present study is avoiding the use of sodium pentobarbital overdoses (100 and 150 mg/kg) during euthanasia protocol because they can interfere with most biochemical, molecular and histological measurements.

## Materials and Methods

### Experimental animals

Male and female Wistar (outbred strain) rats, weighing 150–160 ± 5 g. (National Research Center, Dokki, Egypt) were used in this study. During the acclimation period (one week), standard rodent food pellets (Agricultural-Industrial Integration Company, Giza, Egypt) and tap water (2 bottles fitted in each cage) were provided *ad libitum*. Rats were grouped and housed in polyacrylic cages (six animals per cage) and supplied with bedding (saw dust) and nesting (Kleenex tissues) material. The room was maintained under a 12:12 h light: dark schedule with the white light on between 02:00 and 14:00 h and continuous dim red light (two 60 watt bulbs, Serma Electrical, Cairo, Egypt) enabling observation during the dark period, and at a constant temperature (22–25 °C). Experimental protocols and procedures used in this study were approved by the Cairo University Institutional Animal Care and Use Committee (CU-IACUC) (Egypt), (approval no. CU/I/F/86/18) in accordance with the international guidelines for care and use of laboratory animals.

### Experimental design

Thirty six rats (18 male and 18 female) were assigned into three main groups (6 rats/group) as follows:

***Group I****.* Rats of this group were injected intraperitoneally with 50 mg/kg sodium pentobarbital (2.5 ml/kg).

***Group II****.* Rats of this group were injected intraperitoneally with 100 mg/kg sodium pentobarbital (2.5 ml/kg).

***Group III****.* Rats of this group were injected intraperitoneally with 150 mg/kg sodium pentobarbital (2.5 ml/kg).

All animals were immediately euthanized by carotid exsanguination just after loss of consciousness (2.21 ± 0.35 min. for 50 mg/kg and 1.1 ± 0.24 min. for 100 &150 mg/kg). Blood was collected in EDTA tubes (4 ml/rat) while liver, kidney and spleen of the rats were quickly removed and immediately divided into two portions. Part of each organ was immediately placed in 10% (v/v) formal saline for histological examination and the rest was kept at −80 °C for subsequent assays.

### Sample preparation

Blood samples collected in centrifuge tubes were centrifuged at 3000 rpm (RCF = 1008 × g) for 20 minutes. Serum was stored at −20 °C until used for biochemical assays. Liver organ was homogenized (10% w/v) in ice-cold 0.1 M Tris-HCl buffer (pH 7.4). The homogenate was centrifuged at 3000 rpm for 15 min. at 4 °C and the resultant supernatant was used for biochemical analysis.

### Reverse transcription-quantitative polymerase chain reaction (RT-qPCR)

Total RNA was extracted from frozen samples of liver and kidney organs from male and female rats injected by the three different doses of sodium pentobarbital (6 rats/group); 50 mg/kg, 100 mg/kg and 150 mg/kg using the GeneJET RNA Purification kit (Thermo Fisher Scientific, Inc., Waltham, MA, USA), according to the manufacturer’s instructions and was stored at −80 °C. First strand cDNA was prepared from 1 μg of total RNA using Maxima First Strand cDNA synthesis kit (Thermo Fisher Scientific, USA), according to the manufacturer’s instructions. Real-time PCR was performed to quantify the synthesized cDNA using Maxima SYBR-Green Master Mix kit (Thermo Fisher Scientific, USA) for amplification of Hif1a and Tnfa genes. The reaction was detected with Applied Biosystem 7500 Step One Plus using glyceraldehyde 3-phosphate dehydrogenase (GAPDH) as a housekeeping control gene^57^. Each sample was prepared as duplicate for each gene. Primers used for qPCR were commercially synthesized by Macrogen, Inc. (Seoul, Korea) (Table 3). Total volume for each qPCR reaction was 25 µl. Each sample was initially denatured at 95 °C for 5 min, and then was subjected to 40 cycles of denaturation at 95 °C for 50 sec, annealing and extension at 60 °C for 1 min, then final extension at 72 °C for 10 min. Melting curves were also conducted after amplification to ensure the reaction specificity. Following qPCR, Cq values were detected and used to calculate ΔΔCq and fold expression. Results are reported as Mean ± Standard Error (SE) of relative change compared to the control group (treated with 50 mg/kg of sodium pentobarbital).

**Table 3:** Primer sequences for Hif1a, Tnfa and GAPDH genes in rats used for RT-qPCR.

Gene	Sequence
Hif1a	F: 5′AGAACTCTCAGCCACAGTGC 3′
	R: 5′CAGAAGGACTTGCTGGCTGA3′
Tnfa	F: 5′CGTCAGCCGATTTGCCATTT3′
	R: 5′TCCAGTGAGTTCCGAAAGCC3′
GAPDH	F: 5′AGTGCCAGCCTCGTCTCATA3′
	R: 5′GATGGTGATGGGTTTCCCGT3′

### Histological and histochemical techniques

Samples were taken from liver, kidney, and spleen from male and female Wistar rats and were fixed in 10% formal saline for further routine processing: dehydration, clearing, and embedding. The paraffin embedded blocks of tissues were sectioned using microtome into 4 μm -thick sections. Tissue sections on slides were dewaxed, hydrated, stained by hematoxylin and eosin stains, dehydrated, cleared and mounted for light microscopic examination^58^.

### Immunohistochemical examination

De-paraffinized and hydrated sections from hepatic and renal organs were rinsed with PBS for 15 min. The sections were blocked with normal goat serum (1.5% in PBS) and then incubated (45 min, room temperature) with HIF1-alpha antibody specific for the active form (1.0 μg/ml). Then, the sections were incubated with gold (1 nm)-conjugated goat anti-rabbit IgG (1:200; 30 min, room temperature) and developed with silver enhancement solution (Amersham Pharmacia Biotech silver enhancement system) for 5 min. Sections were counterstained with methyl green. For negative controls, rabbit IgG (1 μg/ml) instead of the primary antibody was added to the reaction. Finally, the tissue sections were counterstained with hematoxylin and mounted using DPX and were examined using light microscope (Olympus, CX41, Japan). The percentage of surface area expressed by HIF1-alpha in liver and kidney sections from different groups was assessed using 3 sections for each slide. At least 15 fields per section were accounted using CellSens dimensions software (Olympus).

### Serum biomarkers for liver function tests and total protein level

The appropriate kits (Bio-Diagnostic, Dokki, Giza, Egypt) were used for the determination of serum aminotransferase enzyme activities (AST&ALT). Serum glucose concentration was measured by the glucose oxidase method^59^. Urea was determined using colorimetric end point method according to modified bromocresol green binding assay (BCG)^60^. Creatinine in the serum was determined by colorimetric method according to Tietz (1986)^61,62^.

### Oxidative stress markers assessment

Oxidative stress markers were detected in the resultant supernatant of liver homogenate. The appropriate kits (Biodiagnostic kits, BiodiagnosticDokki, Giza, Egypt) were used for the determination of malondialdehyde (MDA)^63^, reduced glutathione (GSH)^64^, and catalase (CAT)^65^.

### Statistical analysis

SPSS (version18.0) was used for the statistical analysis. All values were expressed as means ± SE. Student *t* test or Two way analysis of variance (ANOVA) with the Duncan post hoc test was used to compare the genes expressions, the percentage area of positive reaction in IHC and other biochemical parameters (AST, ALT, glucose, urea, creatinine, MDA, GSH and catalase) between groups. P -value ≤ 0.05 was considered statistically significant.

## Data Availability

All data generated or analyzed during this study are included in this published article.
